# Janus Microgel Robots for Actively Boosting Catalytic Efficiency and Recovery of Living Materials

**DOI:** 10.34133/research.1167

**Published:** 2026-02-25

**Authors:** Changming Lan, Jing Liang, Jie Xu, Xinxin Wang, Xiao Liu, Yiran Ni, Yingnan Zhou, Ming-Bang Wu, Chao Zhang, Junqiu Liu, Baiheng Wu

**Affiliations:** ^1^ Key Laboratory of Organosilicon Chemistry and Material Technology, Ministry of Education, Zhejiang Key Laboratory of Organosilicon Material Technology, College of Material, Chemistry and Chemical Engineering, Hangzhou Normal University, Hangzhou 311121, China.; ^2^ College of Chemical and Biological Engineering, Zhejiang University, Hangzhou 310027, China.; ^3^ School of Materials Science and Engineering, Zhejiang Sci-Tech University, Hangzhou 310018, China.; ^4^ MOE Key Laboratory of Macromolecular Synthesis and Functionalization, and Key Laboratory of Adsorption and Separation Materials & Technologies of Zhejiang Province, Department of Polymer Science and Engineering, Zhejiang University, Hangzhou 310027, China.

## Abstract

Leveraging living materials such as algae as sustainable photocatalytic platform is highly promising for mitigating antibiotic water pollution; however, they are confronted with low catalytic efficiency and difficulty in recovery, as imposed by their passive, static working mode. Herein, we report a Janus microgel robot (JMR) that features the integration of magnetic-controlled mobility and living photocatalytic function, allowing for substantially boosting antibiotic degradation efficiency and efficient recovery in an actively magnetic-controlled manner. The key to the JMR lies in harnessing gas shear microfluidic technique to manipulate the spatial distribution of TiO_2_–*Chlorella pyrenoidosa* and Fe_3_O_4_ phases into a Janus architecture, followed by gel encapsulation to prevent cell leakage. Under simulated sunlight, the JMR system achieves 77% antibiotic degradation within 10 h, which is 10 times that of the free *C. pyrenoidosa* (7.6%). Moreover, we demonstrate that the JMR can be imparted with enhanced degradation efficiency by 10.6% and over 95% effectiveness through 3 consecutive operational cycles in actively magnetic-controlled mode. This work establishes a prototype for sustainable environmental biorobots and provides a novel strategy for photocatalytic-biological hybrid system design, advancing the next-generation living materials for water treatment.

## Introduction

Antibiotic contamination of aquatic ecosystems presents severe environmental and public health risks, with antibiotic resistance causing over 1 million annual deaths globally [1–4]. World Health Organization projections indicate that this mortality will rise to 1.91 million by 2050, necessitating urgent intervention [5–8]. Current methods for antibiotic removal predominantly involve adsorption and advanced oxidation processes [9,10]. While effective for rapid degradation, these techniques inevitably generate solid waste, causing secondary environmental pollution [11]. In contrast, microbial degradation via biological treatment offers advantages such as environmental compatibility and lower economic costs [12]. Microalgae, as primary producers in aquatic ecosystems, demonstrate particular promise for aquatic antibiotic remediation due to rapid growth cycles, high pollutant sensitivity, and innate degradative capabilities [13–15]. However, practical implementation remains constrained by low degradation efficiency and poor recoverability of suspended microalgal cells [16–18], highlighting the need for integrated systems combining enhanced processing capacity with facile recyclability.

Aiming at the challenges of limited degradation efficiency and difficult recovery of suspended microalgal cells, the field of engineering living materials proposes to integrate living cells with functional synthetic components to prepare advanced biological hybrid systems [19,20]. Among them, the living microrobot is the most representative. Living microrobots use cells as living primitives and integrate motion function modules such as magnetism to realize the function of materials in precise space and efficient recovery after the task is completed [21,22]. For instance, a magnetic microalgae robot has manifest the potential to actively remove plastics in the environment and achieve simple magnetic recovery [23]. In addition to the active working mode, the living components can also be functionally enhanced, as beautifully validated by improved hydrogen production performance of microalgae through biomineralization strategy. Such strategies can be seamlessly integrated into the microrobot architecture to improve its repair performance to the environment [24]. Therefore, the microrobot strategy combined with the biological function enhancement strategy paves the way for the development of a new generation of microrobots, which is expected to become an integrated and sustainable environmental remediation method for efficient degradation and closed-loop recovery of antibiotic pollutants in aquatic environment.

**Fig. 1.**
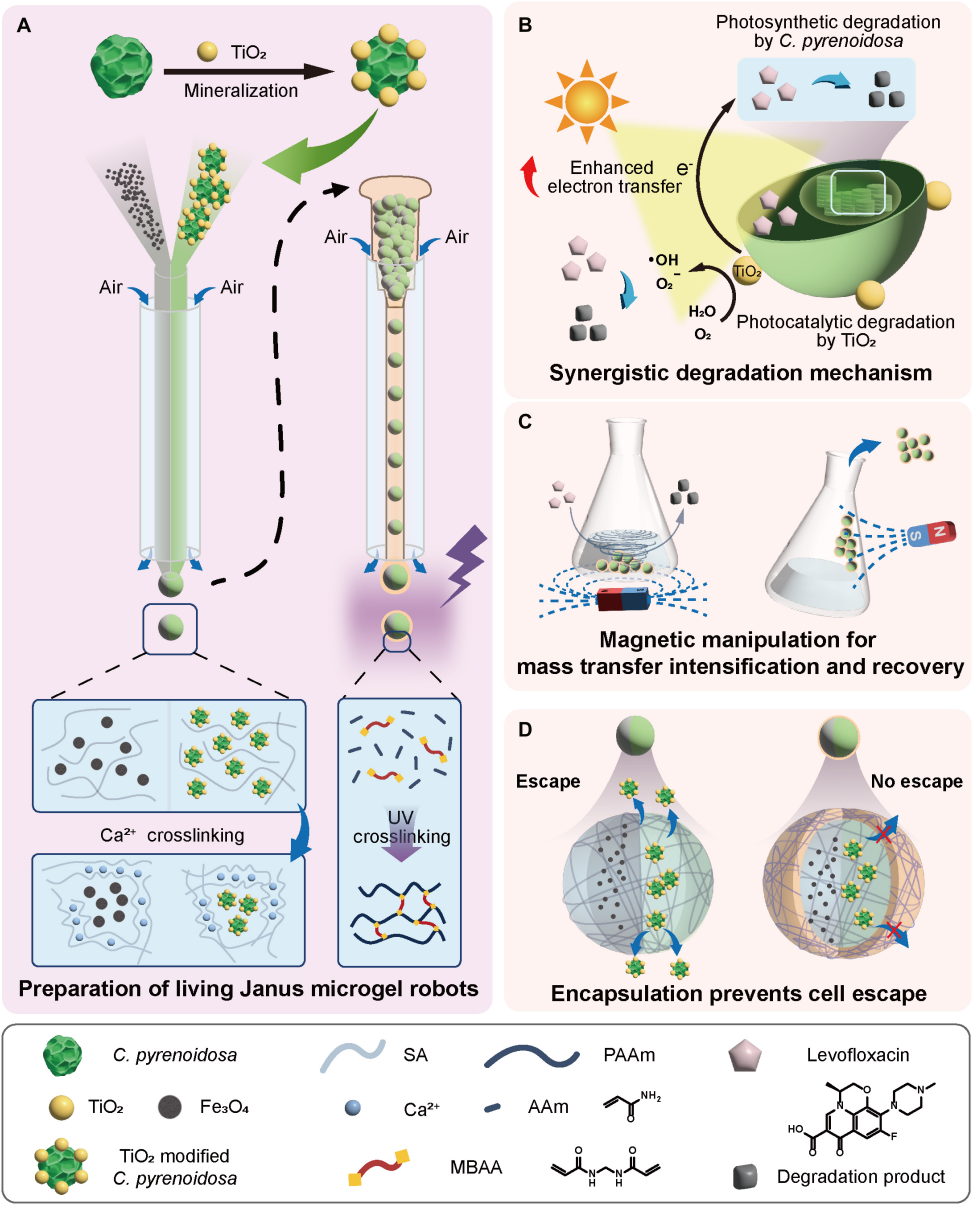
Schematic illustration of the JMRs. (A) Mineralization of TiO_2_–*C. pyrenoidosa* and construction of JMRs by gas shear microfluidic technique. (B) Synergistic degradation mechanism of JMRs. (C) Schematic illustration of the accelerated mass transfer and recovery of JMRs under magnetic field. (D) Schematic illustration of the secondary encapsulation of JMRs to prevent cell escape.

Here, we discover Janus microgel robots (JMRs) by a multi-scale engineering approach integrating TiO_2_ biomineralization on *Chlorella pyrenoidosa* with gas-shearing microfluidic approach as illustrated in Fig. 1A. At the subcellular/cellular scale, mineralized TiO_2_ nanoparticles donate photogenerated electrons to enhance photosynthetic electron transport from PSII to PSI, synergistically accelerating antibiotic metabolism while enabling photocatalytic degradation (Fig. 1B). Above the cellular scale, microfluidically structured JMRs colocalize biohybrids and Fe_3_O_4_ nanoparticles within discrete hemispheric compartments. The TiO_2_–*C. pyrenoidosa* hemisphere maintains an optimized microenvironment for photodegradation, while the Fe_3_O_4_ nanoparticles hemisphere enables both intensified mass transfer via rotational magnetic actuation and targeted recovery (Fig. 1C). Subsequent ultraviolet (UV)-initiated polymerization creates a polyacrylamide encapsulation shell preventing biological leakage while preserving photodegradation functionality (Fig. 1D). This integrated design enables the JMR system to degrade 30 mg/l levofloxacin (LEV), representing a 10-fold efficiency increase over free microalgae (requiring >20 d for 10 mg/l). Our work establishes a scalable prototype for sustainable water purification, advancing photocatalytic-biological hybrid systems through multi-scale living material engineering.

## Results and Discussion

### The mineralization of *C. pyrenoidosa*

**Fig. 2.**
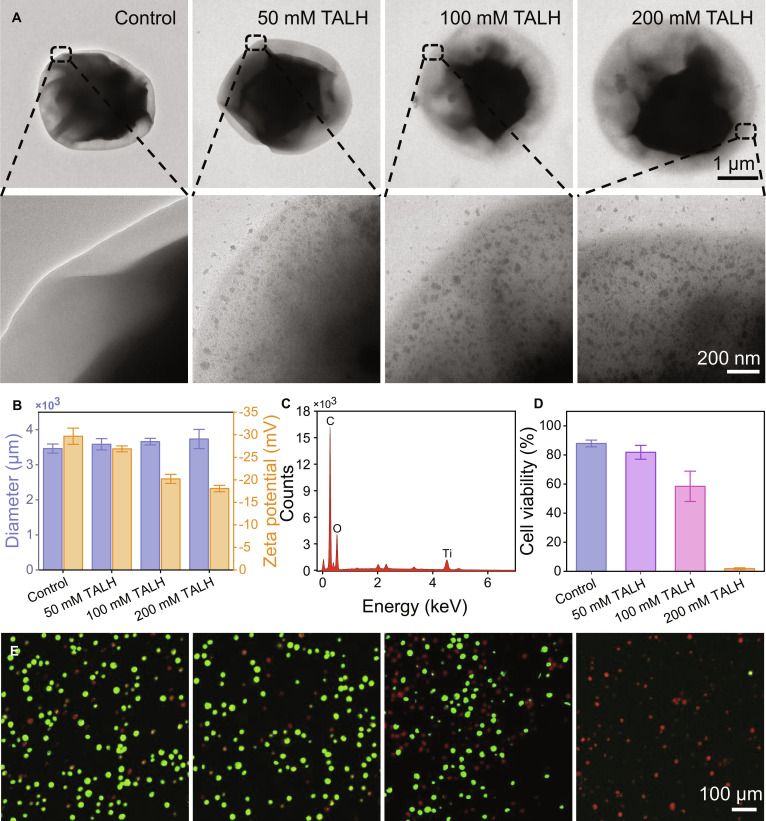
Characterization of TiO_2_–*C. pyrenoidosa* biohybrids. (A) Representative transmission electron microscopy images of *C. pyrenoidosa* cocultured with 0, 50, 100, and 200 mM TALH for 12 h. (B) Size and zeta potential of the biohybrids. (C) Elemental distribution of the 50 mM TALH cocultured group. (D) Quantification and (E) representative confocal laser scanning microscopy images of cell viability across different groups.

The mineralization of semiconductive nanomaterials on microorganisms has been proved to have considerable potential for enhancing metabolic performance in bioproduction and bioremediation applications [25]. In this work, we introduced TiO_2_ to *C. pyrenoidosa* to augment electron transport efficiency and boost bioremediation capabilities. Through controlled surface mineralization via 12-h coculturing with dihydroxy bis(ammonium lactato) titanium(IV) (TALH), a stable TiO_2_–*C. pyrenoidosa* biohybrid system was successfully established. Transmission electron microscopy revealed that TiO_2_ nanoparticle density positively correlated with the concentration of TALH from 0 to 200 mM (Fig. 2A). Dynamic light scattering data showed that with the increase of TALH concentration, the particle size of TiO_2_–*C. pyrenoidosa* gradually increased from 3.4 to 3.7 μm, and the zeta potential positively shifted from −29.7 to −18.1 mV, indicating that TiO_2_ stabilized and bound to microalgae through surface charge interaction (Fig. 2B). Energy-dispersive x-ray spectroscopy confirmed successful biohybrid formation, detecting 22.1% titanium content in the 50 mM TALH group (Fig. 2C and Table S1). Critical biocompatibility assessment demonstrated concentration-dependent viability effects where 200 mM TALH severely reduced cellular viability to 2%. In contrast, microalgae treated with 50 mM TALH exhibited viability equivalent to untreated controls. This concentration achieved optimal balance between mineralization efficiency and cellular vitality and was consequently selected for biohybrid fabrication for subsequent experiments.

### Microfluidic preparation of JMRs

**Fig. 3.**
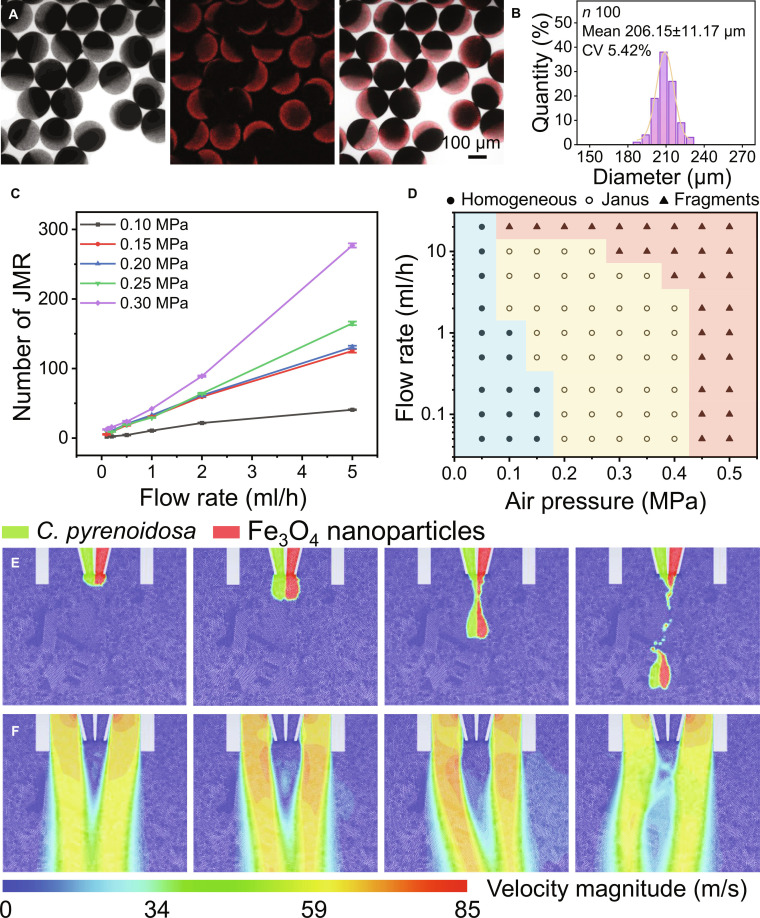
Preparation and structural optimization of JMRs. (A) Representative laser scanning confocal microscope of JMRs prepared at 0.3 MPa air shearing and 2 ml/h of pregel flow rate and (B) corresponding size distribution histogram. (C) Droplet generation frequency quantified over 5-s intervals under varied operational parameters. (D) Phase diagram correlating microgel morphology with gas pressure and flow rate. Fluid dynamic simulation of (E) morphological transitions and (F) velocity field during droplet formation.

Following mineralization, the TiO_2_–*C. pyrenoidosa* biohybrid was further engineered at a macroscopic scale to fabricate living JMRs via gas-shearing microfluidics (Fig. 1A). In this process, 2 distinct alginate-based pregel solutions, containing the TiO_2_–*C. pyrenoidosa* biohybrid and Fe_3_O_4_ nanoparticles, were co-injected into a partitioned glass capillary using syringe pumps. Simultaneously, compressed air flowed through the annular space between the capillary and outer glass tube (Fig. S1), generating shear forces that fragmented the extruded pregel into biphasic droplets. These droplets were subsequently cross-linked in CaCl_2_ solution to form stable microgel with spatially segregated functional domains. Confocal and optical microscopy confirmed well-defined Janus morphology (Fig. 3A). Precise control over microgel size was achieved through operational parameters, yielding an average diameter of 206 μm with narrow size distribution at 0.3 MPa gas pressure and 1 ml/h pregel flow rate (Fig. 3B). Both generation frequency and size exhibited broad tunability (Fig. 3C and Fig. S2). Notably, droplet generation frequency consistently increased with higher pregel solution flow rates across all pressure conditions, demonstrating liquid flow rate as the dominant control factor. Concurrently, elevated air flow rates enhanced production efficiency and reduced microgel size, thereby increasing generation frequency at fixed flow rates, which was validated by high-speed camera images (Fig. S3).

Although Fe_3_O_4_ exhibits high biocompatibility with microalgae at concentrations below 750 mg/l [26], it inhibits microalgal cell growth in this system where the concentration is set at 2,000 mg/l to ensure magnetic motion (Fig. S4). Therefore, a Janus structure with a dual-sided separation design is essential to prevent the adverse effects of such high Fe_3_O_4_ concentrations on microalgae. Thus, we further explore the boundary condition to prepare the well-defined Janus structured microgels. A comprehensive phase diagram was summarized revealing 3 distinct configurations across experimental conditions: homogeneous mixtures, Janus microgels, and fragmented structures (Fig. 3D and Fig. S5). In order to clarify the formation mechanism of JMRs in gas shear microfluidic technology, we have established a force balance model as shown in Fig. S6 and well discussed it in detail in the Supplementary Materials. In short, under low shear conditions, homogeneous mixtures form due to prolonged droplet coalescence, while excessive shear induces fragmentation via Plateau–Rayleigh instability. Well-defined Janus microgels are generated exclusively under moderate shear conditions. In addition, the computational simulations further validated the formation process of Janus droplets, homogeneous mixtures, and fragmented structures (Fig. 3E and Figs. S7 and S8). The velocity field visualization confirmed that the process is highly shear-dependent by showing pronounced difference in velocity gradient (Fig. 3F and Figs. S7 and S8).

### Magnetic manipulation of JMRs

**Fig. 4.**
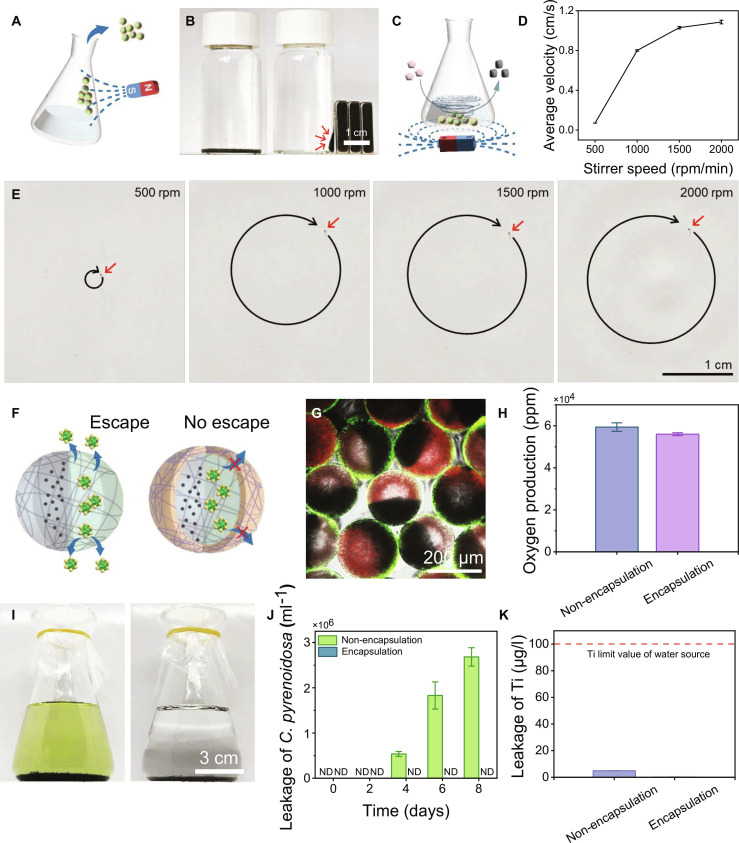
Magnetic manipulation and encapsulation of JMRs. (A) Schematic illustration of magnet recovery of JMRs. (B) Digital images of aggregated JMRs attracted by magnets. (C) Schematic diagram of rotational magnetic field intensified mass transfer. (D) Average movement rate of JMR at different magnetic stirring frequency. (E) Trajectory profiles of JMRs at varying stirring frequency. (F) Illustration of encapsulation of JMRs preventing TiO_2_–*C. pyrenoidosa* escape. (G) Confocal microscopy image revealing the core–shell structure of JMRs after polyacrylamide encapsulation. (H) Influence of encapsulation on oxygen production of *C. pyrenoidosa* after 3-d culture. (I) Solution clarity comparison between non-encapsulated and encapsulated JMRs after 8-d culture. (J) Quantified escaped cell counts of the 2 groups at 2-d intervals. (K) ICP analysis of titanium leaching after 3-d incubation.

Traditional bioremediation of antibiotic by *C. pyrenoidosa* faces harvesting challenges and suffers from limited mass transfer via free diffusion. Incorporating Fe_3_O_4_ nanoparticles into living Janus microgel confers magnetic manipulation functionality to JMRs, enabling targeted recovery and process intensification for antibiotic substrate mass transfer. Magnetic responsiveness was confirmed by JMR migration toward external magnets as illustrated in Fig. 4A and B, which proved post-utilization recovery possible by magnetic harvesting feasible. Under rotational magnetic fields, a single JMR exhibited predictable circulatory motion with velocity proportional to rotational frequency, reaching 1.08 cm/s at 2,000 rpm (Fig. 4C to E and Movie S1). For multiple JMRs, collective motion generated dynamic degradation clusters achieving synchronized rotation at 10°/s angular velocity, substantially enhancing local hydrodynamic mixing and antibiotic degradation efficiency.

In order to prevent the contents of JMRs from escaping into the environment, we constructed a polyacrylamide protective shell on their outer layer, which allowed only small molecules to pass through and limited the escape of *C. pyrenoidosa* cells (Fig. 4F). Confocal microscopy confirmed that the acrylamide (AAm) shell forms a core–shell structure that effectively confines *C. pyrenoidosa* within JMRs (Fig. 4G). Shell encapsulation demonstrated negligible impact on microalgal viability, with oxygen production rates comparable to free cells over 72 h (Fig. 4H). Crucially, encapsulated JMRs prevented cellular escape and maintained solution clarity after 8-d culture, while unencapsulated controls exhibited substantial microalgal release (2.68 × 10^6^ cells) and media coloration (Fig. 4I and J). These results demonstrated the biosafety of JMRs to avoid algal bloom. In addition, to verify the heavy metal safety of JMRs to the environment, inductively coupled plasma (ICP) analysis further confirmed that the leaching titanium after 3-d culture in phosphate-buffered saline (PBS) buffer was just 0.12 μg/l, substantially below both unencapsulated controls (4.91 μg/l) and Ti limit of water source [100 μg/l, Standard for Environmental Quality of Surface Water (GB 3838-2002), Standardization Administration of China and National Health Commission, Beijing, China, 2002]. After encapsulation, this dual biosafety and heavy metal safety of JMRs enabled subsequent antibiotic treatment applications.

### Degradation performance of LEV by JMRs

**Fig. 5.**
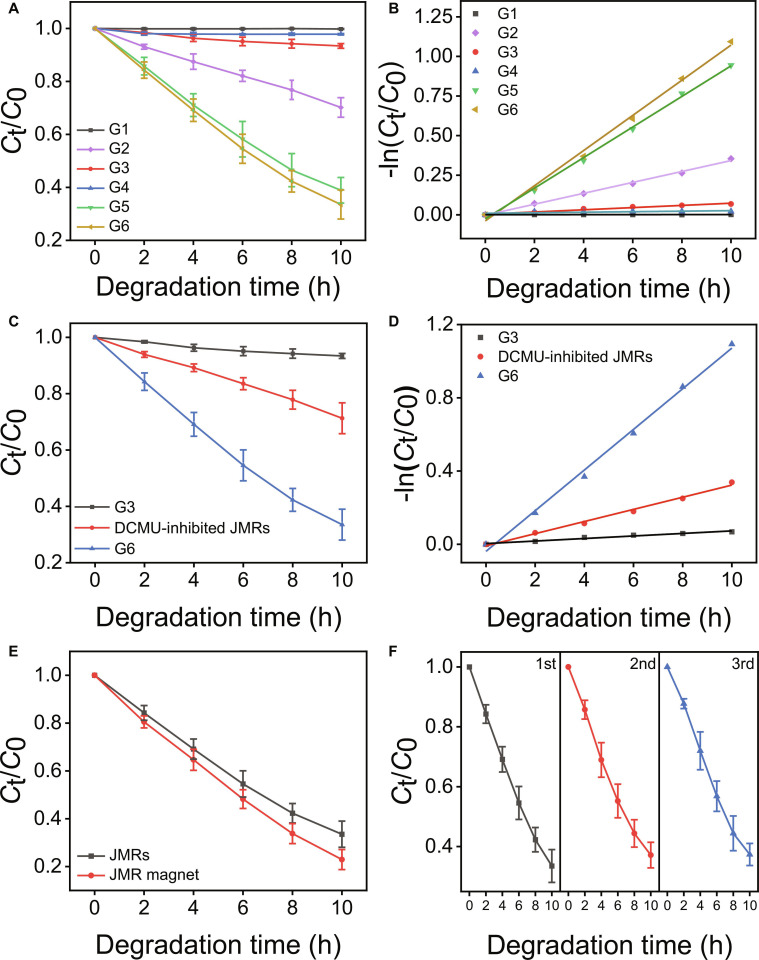
LEV degradation performance by JMRs. (A) Comparative LEV degradation by JMRs and control groups. G1, SA microgel; G2, TiO_2_ nanoparticles; G3, SA/*C. pyrenoidosa* Janus microgel; G4, SA/Fe_3_O_4_ Janus microgel; G5, SA/TiO_2_–*C. pyrenoidosa* Janus microgel; G6, JMRs. (B) Corresponding degradation kinetics from (A). (C) LEV degradation by SA/C*. pyrenoidosa* Janus microgel, JMRs, and DCMU-inhibited JMRs. (D) Corresponding degradation kinetics from (C). (E) Enhanced LEV degradation by JMRs at 2,000 rpm rotating magnetic field. (F) Three consecutive LEV degradation cycles by the same batch of JMRs. All the experiments were conducted at 30 mg/l initial LEV concentration under simulated sunlight.

Building upon the successful fabrication of living JMR materials, the antibiotic degradation performance was studied in detail using LEV as a model system. LEV degradation kinetics by sodium alginate (SA) microgel (G1), TiO_2_ nanoparticles (G2), SA/*C. pyrenoidosa* Janus microgel (G3), SA/Fe_3_O_4_ Janus microgel (G4), SA/TiO_2_–*C. pyrenoidosa* Janus microgel (G5), and JMRs (G6) were systematically evaluated under simulated sunlight at 30 mg/l LEV initial concentration within 10 h as shown in Fig. 5A and B. LEV in G1 and G4 groups exhibited minimal degradation, excluding absorption of SA matrices and confirming inertness of Fe_3_O_4_ nanoparticles. In addition, dark control experiments confirmed negligible contributions from adsorption and non-photoactive biological processes to antibiotic removal (Fig. S9). Crucially, TiO_2_–*C. pyrenoidosa* biohybrids in G5 and G6 demonstrated near-equivalent efficacy (*C*_t_/*C*_0_ = 0.389 and 0.335, respectively), substantially exceeding the additive performance of isolated components as shown by TiO_2_ in G2 (*C*_t_/*C*_0_ = 0.702) and *C. pyrenoidosa* in G3 (*C*_t_/*C*_0_ = 0.934). The *C. pyrenoidosa* mineralized with TiO_2_ showed better degradation ability than ordinary microalgae and TiO_2_, which indicated that there was electronic synergy between them [27]. Mechanistic validation using 30-min exposure to the photosystem II inhibitor dichlorophenyl dimethyl urea (DCMU) further confirmed synergistic electron transfer, suppressing LEV removal efficiency to *C*_t_/*C*_0_ = 0.713 after 10 h (Fig. 5C). This acute inhibition response, occurring markedly faster than the 72-h threshold for DCMU-induced biomass [28], demonstrated specific blockade of the electron transport chain rather than generalized cytotoxicity. The attenuation exceeded intrinsic photosynthetic contributions (G3, *C*_t_/*C*_0_ = 0.934) while approaching TiO_2_-only performance (G2, *C*_t_/*C*_0_ = 0.702), directly implicating photosynthetic electron transport in antibiotic degradation. The kinetic analysis further corroborated the TiO_2_–*C. pyrenoidosa* electronic synergy (Fig. 5D). In addition, cell viability within the JMR system was monitored throughout experiments (Fig. S10). Microalgal activity remained comparable to controls at all stages, confirming robust viability in the JMR environment. To assess potential oxidative damage from reactive oxygen species (ROS) produced by TiO_2_, relative intracellular ROS level and oxidative stress were measured in Fig. S11. The results demonstrated no obvious oxidative damage to microalgal cells during degradation despite measurable ROS. Collectively, these results establish definitive evidence for the hypothesized photocatalytic-photosynthetic synergy within the engineered biohybrids.

In order to further improve the degradation efficiency, we intensified the LEV mass transfer by applying rotational magnetic fields. At 2,000 rpm rotating speed, the *C*_t_/*C*_0_ value of JMRs decreased to 0.229 after 10 h, and the degradation efficiency was increased by 10.6% (Fig. 5E and Fig. S12), which was attributed to the accelerated antibiotic diffusion. The JMRs demonstrated superior degradation performance compared to existing living photodegradation systems, as summarized in Table s2. In addition, the sustainable utilization of JMRs was investigated. The same batch of JMRs was magnetic recycled for 3 times. Through 3 consecutive degradation cycles, JMRs retained >95% initial efficiency. Further validation in Fig. S13 demonstrated that JMRs maintained >85% initial degradation efficiency after 10 cycles, confirming sustained high activity of *C. pyrenoidosa*.

### LEV degradation mechanism and pathway by JMRs

**Fig. 6.**
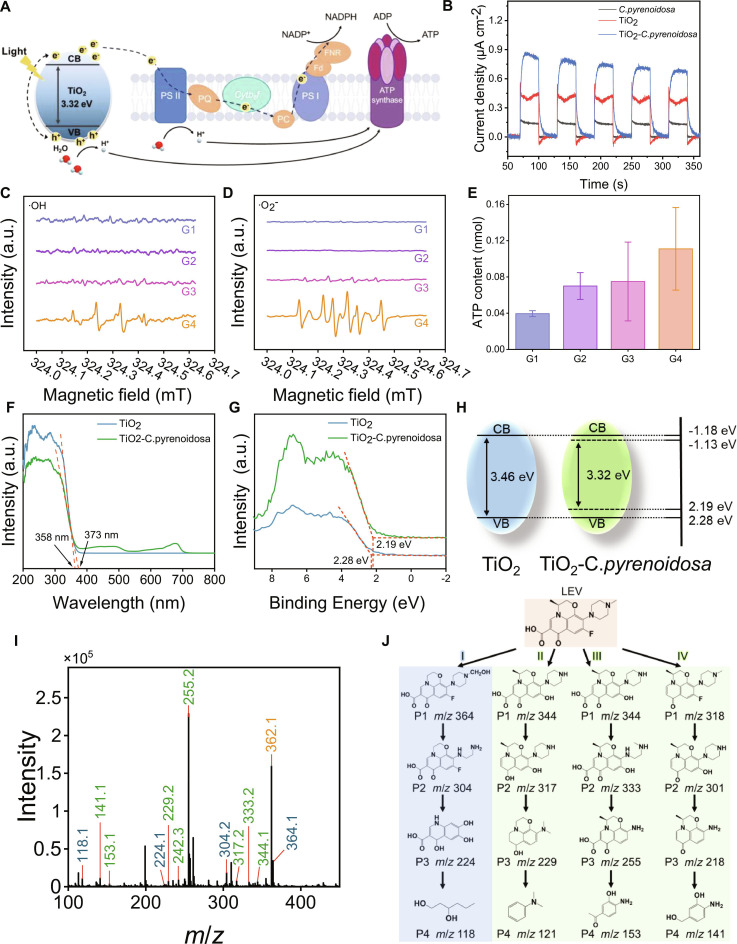
The LEV degradation mechanism and pathway by JMRs. (A) Schematic illustration of photoexcited electron transfer from TiO_2_ to *C. pyrenoidosa*. (B) Transient photocurrent density of *C. pyrenoidosa*, TiO_2_, and TiO_2_–*C. pyrenoidosa*. (C and D) ESR spectra detecting hydroxyl radicals and superoxide anion radicals, respectively. *C. pyrenoidosa* and TiO_2_–*C. pyrenoidosa* in dark condition (G1 and G3) and under illumination (G2 and G4) were studied. (E) ATP content comparison for groups G1 to G4. (F) UV–Vis diffuse reflectance spectra of *C. pyrenoidosa* and TiO_2_–*C. pyrenoidosa*. (G) X-ray photoelectron spectra of *C. pyrenoidosa* and TiO_2_–*C. pyrenoidosa*. (H) Band structure alignment of TiO_2_ and TiO_2_–*C. pyrenoidosa*. (I) LC-MS identification and (J) proposed pathway of LEV degradation intermediates by JMRs.

The efficient degradation of LEV by JMRs arises from synergistic integration of photosynthetic degradation by *C. pyrenoidosa*, which degrade organic compounds by cellular metabolism, and photocatalytic degradation by TiO_2_, which produce hydroxyl radicals and superoxide anion radicals to decompose antibiotics [29]. The synergistic effect was speculated that when TiO_2_ is irradiated by sunlight to produce electrons and holes, some holes will react with water to produce hydroxyl radicals, and some electrons will be captured by thylakoids and participate in the electron transport chain of photosynthesis system of *C. pyrenoidosa* (Fig. 6A) [30,31]. Photoelectrochemical analysis was conducted to verify these speculations. The biomineralization of TiO_2_ on *C. pyrenoidosa* revealed a 0.67 μA cm^−2^ increase in photocurrent density for TiO_2_–*C. pyrenoidosa* biohybrid system (from 0.18 to 0.85 μA cm^−2^) (Fig. 6B). This enhancement indicated that TiO_2_ nanoparticles establish a stable conductive interface that facilitates photogenerated electron transfer, potentially through partial cellular internalization [32,33]. The results of electron spin resonance (ESR) spectroscopy confirmed the exclusive generation of hydroxyl radicals and superoxide anions in TiO_2_–*C. pyrenoidosa* biohybrid under illumination, with no ROS detected in dark conditions or pure microalgal controls (Fig. 6C and D). Critically, pristine TiO_2_ exhibited substantially weaker ROS signals than the biohybrid under identical irradiation (Fig. S14), indicating that microalgae integration enhances interfacial charge separation and boosts radical generation efficiency, directly supporting the photocatalytic LEV degradation pathway.

The energy metabolism in TiO_2_–*C. pyrenoidosa* was studied using ATP (adenosine triphosphate) kit and NADPH [reduced form of nicotinamide adenine dinucleotide phosphate (NADP^+^)] kit to further study the synergistic electron transfer mechanisms in photosynthesis. The markedly elevated ATP and NADPH production of TiO_2_–*C. pyrenoidosa* biohybrid under illumination, persisting even in dark conditions, indicated that photosynthetic metabolic capacity is enhanced (Fig. 6E and Fig. S15). Under simulated sunlight, the photogenerated electrons are injected from TiO_2_ into the redox network of the algae cells, disturbing their redox equilibrium. To balance the excessive reduction tendency, the cells enhance the cyclic photosynthetic phosphorylation or mitochondrial oxidative phosphorylation, using the electron flow to establish a proton gradient to drive ATP synthesis, thereby increasing ATP production [34,35]. At the same time, the photogenerated electrons from TiO_2_ are integrated into the natural photosynthetic electron transport chain, which increases the yield of NADPH. The synergistic effect of external protons and electrons substantially improves the overall metabolic capacity of photosynthesis. We performed UV–visible (UV–Vis) diffuse reflectance spectroscopy and x-ray photoelectron spectroscopy to elucidate the band structure of the biohybrid to further understand the electron transfer TiO_2_ to the photosynthetic system of *C. pyrenoidosa*. As shown in Fig. 6F, TiO_2_–*C. pyrenoidosa* revealed a 15-nm red-shift in absorption edge compared to pure TiO_2_, extending from 358 to 373 nm. This optical transition corresponded to a bandgap narrowing from 3.46 eV of TiO_2_ to 3.32 eV in the biohybrid based on the empirical equation Eg = 1,240/λg. XPS analysis further confirmed electronic interaction, with Ti binding energy decreasing by 0.09 eV in the TiO_2_–*C. pyrenoidosa* biohybrid from that of pure TiO_2_ (from 2.28 eV to 2.19 eV) (Fig. 6G). Based on the above results, the conduction band gaps of pure TiO_2_ and TiO_2_–*C. pyrenoidosa* biohybrid are calculated to be −1.18 and −1.13 eV, respectively (Fig. 6H). The important point is that the band gap width of the TiO_2_–*C. pyrenoidosa* system is narrower than that of TiO_2_, which further confirms that the photogenerated electron transfer from TiO_2_ to *C. pyrenoidosa* occurs. Coupled with elevated NADPH and ATP metabolic levels, these results demonstrate enhanced photosynthetic capacity through supplementary electron donation.

Photosynthetic degradation by *C. pyrenoidosa* and photocatalytic degradation by TiO_2_ in JMRs have distinct degradation pathways for LEV. Liquid chromatography–mass spectrometry (LC-MS) analysis of intermediates from TiO_2_, *C. pyrenoidosa*, and JMRs confirmed that JMR degradation signatures incorporate characteristic products from both isolated photocatalytic and biological pathways (Fig. 6I and Figs. S16 and S17). These evidences establish 4 primary degradation routes for LEV degradation (Fig. 6J). Route I features TiO_2_ dominated stepwise piperazine ring modifications initiated by hydroxylation forming P1 (*m*/*z* = 364) [36], progressing through ring opening to P2 (*m*/*z* = 304), benzene ring defluorination yielding P3 (*m*/*z* = 224), and terminating in decarboxylation to P4 (*m*/*z* = 118) [37–39]. Routes II, III, and IV are *C. pyrenoidosa*-mediated LEV degradation characterized by quinolone skeleton transformation [40]. Route II involves hydroxylation and dealkylation generating P1 (*m*/*z* = 344), decarboxylation forming P2 (*m*/*z* = 317), piperazine ring opening producing P3 (*m*/*z* = 229), and final degradation to P4 (*m*/*z* = 121). Route III proceeds via defluorination and dealkylation to P2 (*m*/*z* = 333), ring opening with dehydroxylation forming P3 (*m*/*z* = 255), and quinolone-benzene ring cleavage yielding P4 (*m*/*z* = 153). Route IV initiates with direct decarboxylation to P2 (*m*/*z* = 301), undergoes hydroxylation and alkylation, proceeds through piperazine ring opening generating P3 (*m*/*z* = 218), and finally degrades to P4 (*m*/*z* = 141) [41–45]. MS quantification revealed signal enhancement in JMR system relative to isolated components, with marked intensification of microalgae-dominant signatures such as *m*/*z* = 255. These results conclusively demonstrate that the degradation of LEV by JMRs is mainly driven by *C. pyrenoidosa*, aligning with prior evidence of TiO_2_-enhanced photosynthetic electron transfer.

## Conclusion

This study established a multi-scale engineering platform for the preparation of recyclable JMRs, achieving efficient and sustainable degradation of LEV in an active working mode. The breakthrough centers on synergistic integration of semiconductor–microorganism hybridization, spatially organized Janus functionalization, and bioactive encapsulation technologies. The mineralization of TiO_2_ nanoparticles on the surface of *C. pyrenoidosa* constructed a biological hybrid interface, where photogenerated electrons augmented the photosynthetic electron transfer chain, substantially enhancing the enhancing metabolic capacity to degrade antibiotics. Gas-shearing microfluidic technology enabled precise fabrication of JMRs that confined TiO_2_–*C. pyrenoidosa* biohybrid in the optimized photodegradation hemispheres while isolating Fe_3_O_4_ nanoparticles in separate domains. This Janus structure eliminated the cytotoxicity of Fe_3_O_4_ nanoparticles while achieving magnetically enhanced mass transfer and JMR recovery. UV-initiated encapsulation effectively restricted the escape of microalgal cells, ensuring operational biological safety. The integrated JMR system degraded 30 mg/l LEV under simulated sunlight within 10 h, exhibiting 10-fold greater efficiency than free microalgae. Magnetic agitation further accelerated mass transfer, boosting degradation efficiency by 10.6%, and maintaining over 95% effectiveness through 3 consecutive operational cycles, demonstrating exceptional reusability. Beyond providing a scalable prototype for sustainable water purification, this research elucidates the functional coupling mechanism between semiconductor materials and microalgae at the photosynthetic interface, establishing fundamental principles for designing next-generation photocatalytic-biological hybrid living materials.

## Materials and Methods

### Materials

All chemical reagents used do not require further purification. SA (viscosity 200 ± 20 MPa·s), anhydrous calcium chloride (CaCl_2_), AAm, N, N-methylene bisacrylamide (MBAA), phenyl-2,4,6-trimethylbenzoyl lithium phosphinate (LAP), Fe_3_O_4_ nanoparticles, LEV, TALH, DCMU, 5,5-dimethyl-1-pyrroline-n-oxide (DMPO), and fluorescein diacetate (FDA) were purchased from Shanghai Aladdin Biochemical Technology Co. Ltd. The ATP assay kit and NADP^+^/NADPH assay kit were purchased from Shanghai Biyuntian Biotechnology Co. Ltd. The resistivity of high-purity water is 18.2 MΩ.cm, which comes from UNIQUE-R40 instrument.

### Microalgae cultivation

*C. pyrenoidosa* was purchased from Freshwater Algae Culture Collection at the Institute of Hydrobiology. These microalgae were cultured in tris-acetate-phosphate (TAP) medium at 25 °C and in a light incubator (light intensity of 4,000 lx, light/dark cycle of 12/12 h), and the culture medium was shaken every 10 h.

### Construction of TiO_2_–*C. pyrenoidosa* system

In order to construct the TiO_2_–*C. pyrenoidosa* system, *C. pyrenoidosa* in exponential growth phase was centrifuged (3,000 rpm, 5 min) to remove the medium, and washed 3 times with deionized water to remove the residual medium to obtain microalgae cells. The above microalgae cells were prepared into 30-ml algal cell suspension with deionized water, added with 50 mM TALH, and placed in a shaker at a speed of 160 rpm for coculture for 12 h under light conditions and room temperature. Then, the culture medium was removed by a centrifuge (3,000 rpm, 5 min) and washed with deionized water 3 times to remove the residual culture medium to obtain the TiO_2_–*C. pyrenoidosa* system.

### Cell viability assessment

Mineralized algal cells were subjected to gradient dilution. A 1-ml aliquot of the diluted mineralized microalgae suspension was incubated with 10 μl of FDA at 37 °C in the dark for 10 min. Following incubation, cells were washed with sterile PBS and observed under a laser confocal microscope. Mineralized microalgae cells exhibit intrinsic red fluorescence. Viable cells, possessing esterase activity, hydrolyze FDA to fluorescein, resulting in green fluorescence. Cell viability was calculated as the percentage of cells exhibiting green fluorescence relative to the total number of fluorescent cells.

### Preparation of microfluidic chip

The glass capillary was drawn into a pointed glass capillary by a microelectrode control instrument (RWD Life Science MP-500), and then the pointed part was polished with 3,000-mesh sandpaper to an inner diameter of about 200 μm. The polished capillary was placed in the center of a circular glass tube with an inner diameter of about 1 cm, and the microfluidic chip required for gas shear was assembled with glue and needle (Fig. S1).

### Preparation of JMRs

In the preparation of JMRs, 2 solutions were injected in parallel through the independent liquid delivery channel of the microfluidic chip: the mixture of the above mineralized microalgae solution with OD750 (optical density at 750 nm) = 1.5 and 4% (w/v) SA solution, and the mixture of 0.2% (w/v) Fe_3_O_4_ nanoparticles and 4% (w/v) SA solution. The flow rate of microalgae and Fe_3_O_4_ nanoparticles is set to 2 ml/h, and the air is injected into the gap between the partition glass capillary and the outer glass tube by the air pump of the 3-dimensional (3D) printer. The air pressure is set to 0.3 MPa, which provides a good shear force for the formation of microdroplets, so that the droplets can be well cut into spherical droplets without dispersion. In the pressure range of 0.2 to 0.4 MPa, we can control the size of JMRs well. Then, we collected it in a 1% (w/v) calcium ion bath below and finally obtained a cross-linked JMRs.

### Encapsulation of JMRs

By configuring 20% (w/v) AAm and 2% (w/v) MBAA solution into the pregel required for secondary cross-linking, by adding 0.05% (w/v) LAP as an initiator, the pregel required for secondary cross-linking was finally mixed with the collected JMRs, and then pumped into the coaxial microfluidic chip and cut by air to obtain JMRs with a thin AAm shell.

### Detection of leaky microalgae

In order to verify the effectiveness of the secondary encapsulation, we set up the encapsulation group and the non-encapsulation group to encapsulate the same OD of *C. pyrenoidosa* in TAP medium at 25 °C, light intensity of 4,000 lx, and light cycle of 12/12 h under the condition of 8 d. In order to evaluate the leakage of microalgae cells during the culture process, 1 ml of solution samples was collected every 2 d using a sterile pipette and centrifuged at 6,000 rpm for 5 min at 4 °C. After discarding the supernatant, 1 ml of sterile phosphate buffer was added to resuspend the cell precipitate. Finally, the blood cell counting plate was used to detect the leakage of microalgae.

### Detection of leaky Ti

To detect Ti element leakage, encapsulation and non-encapsulation groups mineralized for the same duration and encapsulated with *C. pyrenoidosa* were incubated in sterile PBS for 3 d. The supernatant containing leached Ti was collected, filtered through a 0.45-μm pore-size membrane filter, and subjected to acid hydrolysis pretreatment. Quantification of Ti was subsequently performed using ICP-MS.

### Degradation of LEV

The LEV degradation kinetics of SA microgel (G1), TiO_2_ nanoparticles (G2), SA/*C. pyrenoidosa* Janus microgel (G3), SA/Fe_3_O_4_ Janus microgel (G4), SA/TiO_2_–*C. pyrenoidosa* Janus microgel (G5), and JMRs (G6) were systematically evaluated in a 30 mg/l LEV aqueous solution prepared with deionized water, under simulated sunlight (1,000 μmol·m^−2^·s^−1^) at 25 °C over 10 h. The sample solution was taken every 2 h, and the antibiotic residue was determined by a UV spectrophotometer (Shimadzu UV-2600) (LEV has a strong absorption signal at 287 nm). A group of JMRs treated with electron blocker DCMU for 30 min was set up for degradation experiments, and the degradation conditions were the same as above. In addition, another set of JMRs will be set up to carry out degradation experiments on the magnetic field of a 2,000 rpm/min magnetic stirrer, and the degradation process will be carried out under the same conditions as the above experiments (all *n* values above are 3). In the repeated use experiment, JMRs were placed in 30 mg/l solution for 10 h, and LEV residue was measured every 2 h for 3 times.

### Photocurrent test

The photocurrent test was performed using an electrochemical workstation (CHINA EDUCATION AU-LIGHT CHI600E) in a 10 mM PBS solution electrolyte through a standard 3-electrode system at 0 V bias. Platinum wire and Ag/AgCl (3 M KCl) were used as counter electrode and reference electrode, respectively, and the photocurrent was measured using a 50 mW cm^−2^ light-emitting diode (LED) white light.

### Detection of free radicals

The hydroxyl radicals and superoxide anions of microalgae and TiO_2_–*C. pyrenoidosa* system were detected by ESR spectrometer under light and dark conditions. DMPO was used to capture hydroxyl radicals and superoxide anions.

### ATP and NADPH detection

The ATP content and NADPH content of microalgae and TiO_2_–*C. pyrenoidosa* system were detected by the ATP kit and NADP^+^/NADPH detection kit (WST-8 method).

### UV diffuse reflectance determination

The UV diffuse reflectance spectra of microalgae and TiO_2_–*C. pyrenoidosa* system were obtained using a UV–Vis diffuse reflectance spectrometer (Shimadzu UV-2600).

### XPS test

The photoelectron spectra of microalgae and TiO_2_–*C. pyrenoidosa* system were obtained by XPS test (Thermo Scientific K-Alpha).

### LC-MS detection of LEV intermediate products

The extraction method and LC-MS analysis of LEV intermediates were extracted with anhydrous methanol. First, anhydrous methanol was vortexed with the sample solution for 10 min and centrifuged at 12,000 rpm for 15 min at 4 °C. The supernatant was used for the analysis of LEV in the intermediate product. Then, LEV intermediates were extracted with high-performance liquid chromatography (HPLC)-grade methanol and analyzed by LC-MS (Agilent 1290 UPLC; Agilent 6550 Q-TOF LC/MS) to detect intermediate signals. The chromatographic column was waters BEH C18, the injection volume was 5 μl, and the mobile phase was 0.1% (v/v) formic acid water mixture and acetonitrile, respectively. The elution operation started from 10 % acetonitrile, increased from 30 s to 2 min to 25%, increased to 90% from 2 to 5 min, and maintained for 8 min. Then, it began to return to the initial state, the total gradient time was 13 min, and the flow rate was set to 0.3 ml/min. The ESI^+^ source parameters were set as follows: ion spray voltage 4.0 kV, capillary temperature 350 °C, vaporizer temperature 350 °C, gas flow rate 12 l/min, and high-purity N_2_. The *m*/*z* range of MS scan was set between 50 and 500.

## Data Availability

The data that support the findings of this study are available from the corresponding author upon reasonable request.
