# Supplementary material for: Janus Microgel Robots for Actively Boosting Catalytic Efficiency and Recovery of Living Materials
Source: Research (Wash D C). 2026 Feb 25;9:1167. doi: 10.34133/research.1167 (PMC12932866; doi:10.34133/research.1167)
Supplement: Supplementary 1 — Supplementary Text Tables S1 and S2 Figs. S1 to S17 Movie S1 [file research.1167.f1.zip › SI 20260128.docx]

Supplementary Materials

**Janus Microgel Robots for Actively Boosting Catalytic Efficiency and Recovery of Living Materials**

*Changming Lan, Jing Liang, Jie Xu, Xinxin Wang, Xiao Liu, Yiran Ni, Yingnan Zhou, Mingbang Wu, Chao Zhang,* Junqiu Liu,* Baiheng Wu**

C. Lan, J. Liang, J. Xu, X. Wang, Y. Ni, Y. Zhou, J. Liu, B. Wu

Key Laboratory of Organosilicon Chemistry and Material Technology, Ministry of Education, Zhejiang Key Laboratory of Organosilicon Material Technology, College of Material, Chemistry and Chemical Engineering, Hangzhou Normal University, Hangzhou, 311121 China

Email: junqiuliu@hznu.edu.cn, bhwu@hznu.edu.cn

X. Liu

College of Chemical and Biological Engineering, Zhejiang University, Hangzhou, 310027 China

M. Wu

School of Materials Science and Engineering, Zhejiang Sci-Tech University, 928 Second Avenue, Xiasha Higher Education Park, Hangzhou, 310018 China

C. Zhang

MOE Key Laboratory of Macromolecular Synthesis and Functionalization, and Key Laboratory of Adsorption and Separation Materials & Technologies of Zhejiang Province, Department of Polymer Science and Engineering, Zhejiang University, Hangzhou, 310027 China

Email: zhangchao7@zju.edu.cn

**Supporting Text**

**Discussion of the formation mechanisms of JMRs**

where liquid extrusion pressure $\boldsymbol{F}_{\boldsymbol{i}}$, capillary force $\boldsymbol{F}_{\boldsymbol{c}}$, gravity $\boldsymbol{F}_{\boldsymbol{g}}$, and $\boldsymbol{F}_{\boldsymbol{p}}$ gas shear pressure gas flow pressure govern droplet dynamics (Figure S6). And there are:

$$F_{c max}=\pi rS$$

$$F_{g}=\rho_{w}gV$$

where $S$ is the droplet surface area, $\rho_{w}$ is the liquid density, V is the unit volume flow rate, and $g$ is the gravitational constant. Given the Bond number $\boldsymbol{B}_{\boldsymbol{d}}=\frac{\boldsymbol{\rho g}ⅆ^{\mathbf{2}}}{\mathbf{4}\boldsymbol{r}}$ and the droplet diameter $ⅆ$ substantially below 1 m, gravitational effects are negligible in this system^[1]^.

The boundary conditions for droplet formation are:

$$\boldsymbol{F}_{\boldsymbol{i}}<\boldsymbol{F}_{\boldsymbol{c} \boldsymbol{max}}$$

$$\boldsymbol{F}_{\boldsymbol{P}}+\boldsymbol{F}_{\boldsymbol{i}}>\boldsymbol{F}_{\boldsymbol{c} \boldsymbol{max}}$$

transforming to dimensionless parameters, stable microsphere generation requires a Weber number $\boldsymbol{We}$ exceeding 40:

$$\boldsymbol{We}\mathbf{=}\frac{\boldsymbol{\rho}_{\boldsymbol{g}}\boldsymbol{v}_{\boldsymbol{g}}^{\mathbf{2}}\mathbf{ⅆ}_{\boldsymbol{j}}}{\boldsymbol{r}}$$

$$\boldsymbol{\nu}_{\boldsymbol{g}}\mathbf{=}\left( \frac{\mathbf{2}\boldsymbol{P}}{\boldsymbol{\rho}_{\boldsymbol{g}}} \right)^{\frac{\mathbf{1}}{\mathbf{2}}}$$

Where $\boldsymbol{\rho}_{\boldsymbol{g}}$ is the gas density, $\boldsymbol{P}$ is the airflow pressure (0.1-0.5 MPa), $\mathbf{ⅆ}_{\boldsymbol{j}}$is the characteristic diameter (200-800 μm), and $\boldsymbol{r}$ is the surface tension coefficient (0.068 N/m). Substituting experimental parameters yields $\boldsymbol{We\gg40}$, confirming stable droplet formation feasibility^[2]^.

High gas flow rates induce fragmentation via Plateau-Rayleigh instability. The critical disturbance wavelength $\boldsymbol{\lambda}$ of the cylindrical jet under external axial disturbance satisfies $\boldsymbol{k}\mathbf{=}\frac{\mathbf{2}\boldsymbol{\pi}}{\boldsymbol{\lambda}}\mathbf{=1}$, causing jet breakup and satellite droplet formation. This regime is characterized by the Ohnesorge number $\boldsymbol{Oh}$:

$\boldsymbol{Oh}\mathbf{=}\frac{\boldsymbol{u}}{\sqrt{\boldsymbol{\rho}_{\boldsymbol{g}}\boldsymbol{r}\boldsymbol{d}_{\boldsymbol{j}}}}\mathbf{=}\frac{\sqrt{\boldsymbol{We}}}{\boldsymbol{Ra}}$.

Where $\boldsymbol{Ra}$ is the Reynolds number and $\boldsymbol{u}$ is the viscosity coefficient. At constant $\boldsymbol{Oh}$, increasing $\boldsymbol{We}$, enhances inertial forces, destabilizing jets at smaller cone positions; At low constant $\boldsymbol{We}$, $\boldsymbol{Oh}$ minimally affects breakup length; At high constant $\boldsymbol{We}$, increasing $\boldsymbol{Oh}$ suppresses rupture via viscous forces, eliminating satellite droplets. Therefore, higher $\boldsymbol{Oh}$ promotes droplet stability.

In conclusion, according to previous discussion, homogeneous mixtures formed at low Weber number (We<40), where reduced shear prolongs droplet lifetime, enhancing fluid-driven mixing during collision and deformation^[1-3]^. Fragmented structures emerged at high gas flow rates due to Plateau-Rayleigh instability, producing satellite droplets resulting in non uniform Janus microgels. Well-defined Janus microgels generated when with moderate Ohnesorge number.

**Supporting Table and Figures**

**Table S1.** Comparison of degradation performance of this work with other relevant studies.

| Sample | Antibiotic | | Time (h) | Dosage  (cells/mL) | | Degradation rate (mg/L**/**h) | Reference |
| --- | --- | --- | --- | --- | --- | --- | --- |
| *Haematococcus*^[4]^ | OFL | 12 | | 1.0×10^6^ | 0.664 | | Chem. Eng. J. 2023, 465, 142770 |
| *Platymonas subcordiformis*^[5]^ | ENR | 4 | | 3.5×10^8^ | 0.35 | | Photochem. Photobiol. Sci. 2015, 14, 693-699 |
| *C. pyrenoidosa*^[6]^ | CIP | 48 | | 1.0×10^6^ | 0.02 | | J. Hazard. Mater. 2025, 500, 140558 |
| *C. sorokiana*^[7]^ | CIP | 24 | | 5.0×10^5^ | 0.2 | | J. Hazard. Mater. 2024, 466, 133519 |
| *C. vulgaris*^[8]^ | LEV | 48 | | 1.0×10^6^ | 0.008 | | Chemosphere, 2022, 292, 133438 |
| Microalgae-fungus system^[9]^ | NFX | 72 | | - | 0.01 | | Bioresour. Technol. 2024, 400, 130668 |
| *C. ellipsoidea*-*C. rosea*-MWCNT^[10]^ | CPFX | 240 | | 2.0×10^6^ | .032 | | J. Water Process Eng. 2024, 64, 105683 |
| *S. obliquus*-CeO_2_^[11]^ | SD | 240 | | 7.5×10^4^ | 0.00016 | | Bioresour. Technol.2020, 309, 123452 |
| JMRs | LEV | 10 | | 3.5×10^7^ | 2.34 | | **This work** |

**Table S2.** Elemental analysis results of 50 mM TALH co-cultured *C. pyrenoidosa* by Energy dispersive X-ray spectroscopy.

| Element | Wt% | Wt% Sigma | At% |
| --- | --- | --- | --- |
| C | 49.46 | 0.27 | 64.79 |
| O | 28.41 | 0.25 | 27.94 |
| Ti | 22.13 | 0.28 | 7.27 |
| Total | 100.0 |  | 100.0 |


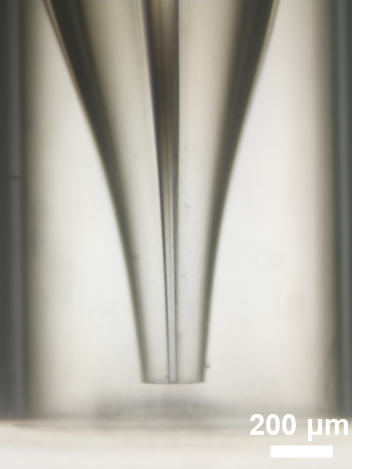


**Figure S1.** Microscope image of the microfluidic chip used to prepared Janus microgel robots.


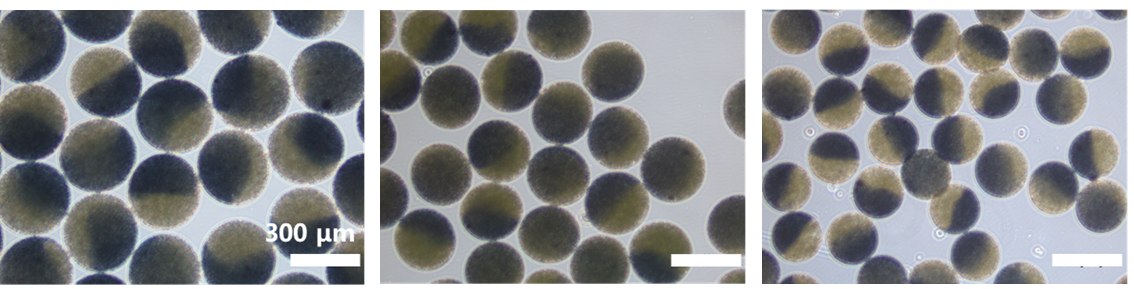


**Figure S2.** Microscopic images of JMRs with different sizes 300 μm, 250 μm and 200 μm obtained at a flow rate of 2 mL/h and shear pressures of 0.2, 0.25, and 0.3 MPa, respectively.


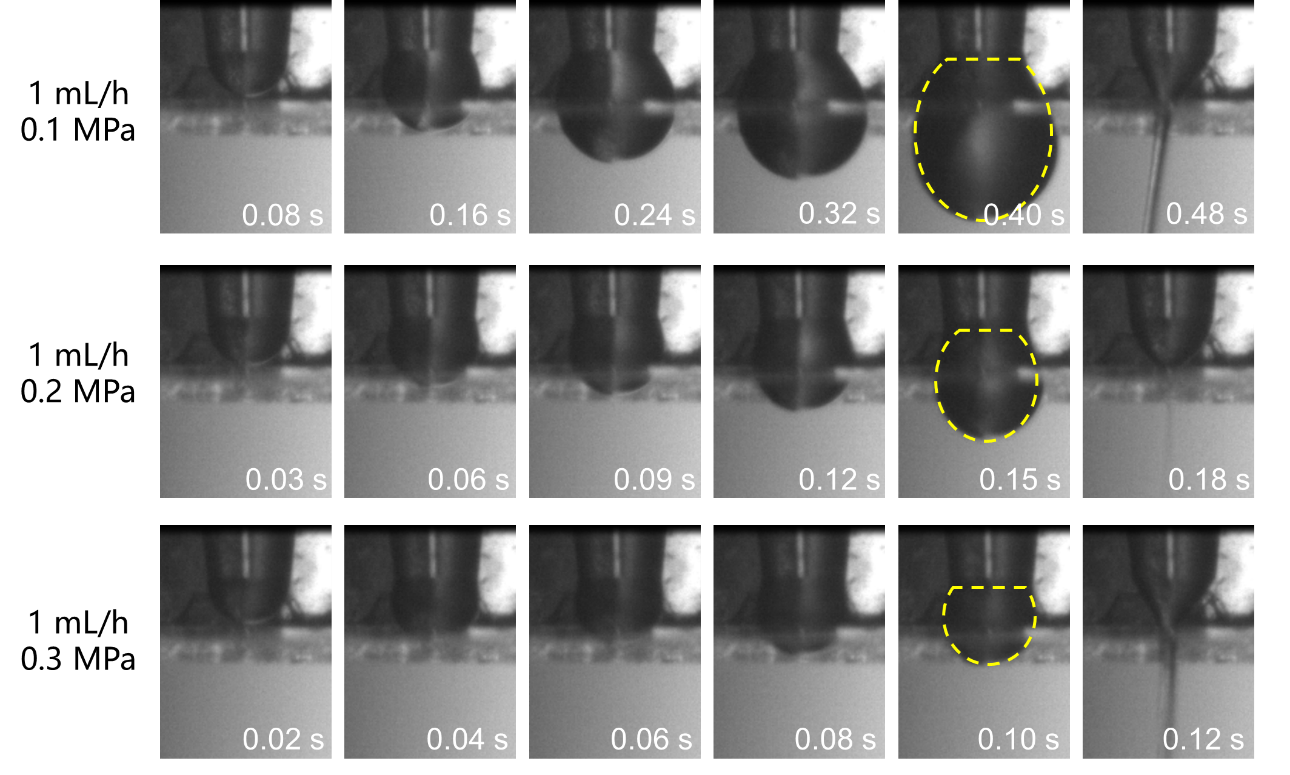


**Figure S3.** High-speed camera captures the droplet formation process under different

shear pressures.

**
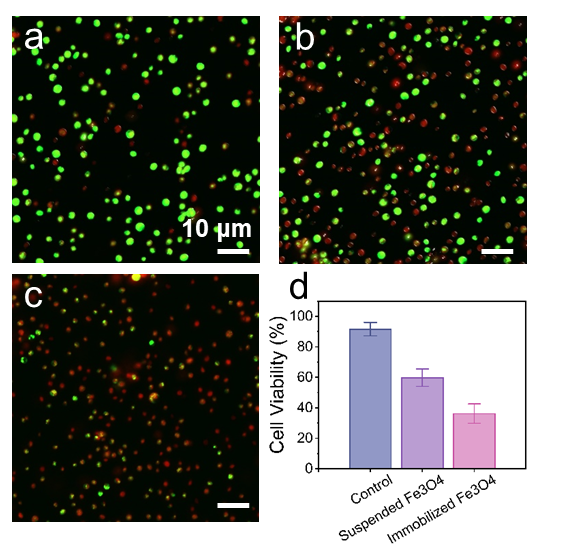
**

**Figure S4.** Viability of *C. pyrenoidosa* cells after 3-day incubation. (a) untreated control, (b) co-cultured with suspended Fe_3_O_4_, and (c) co-cultured with gel-immobilized Fe_3_O_4_ for 3 days.


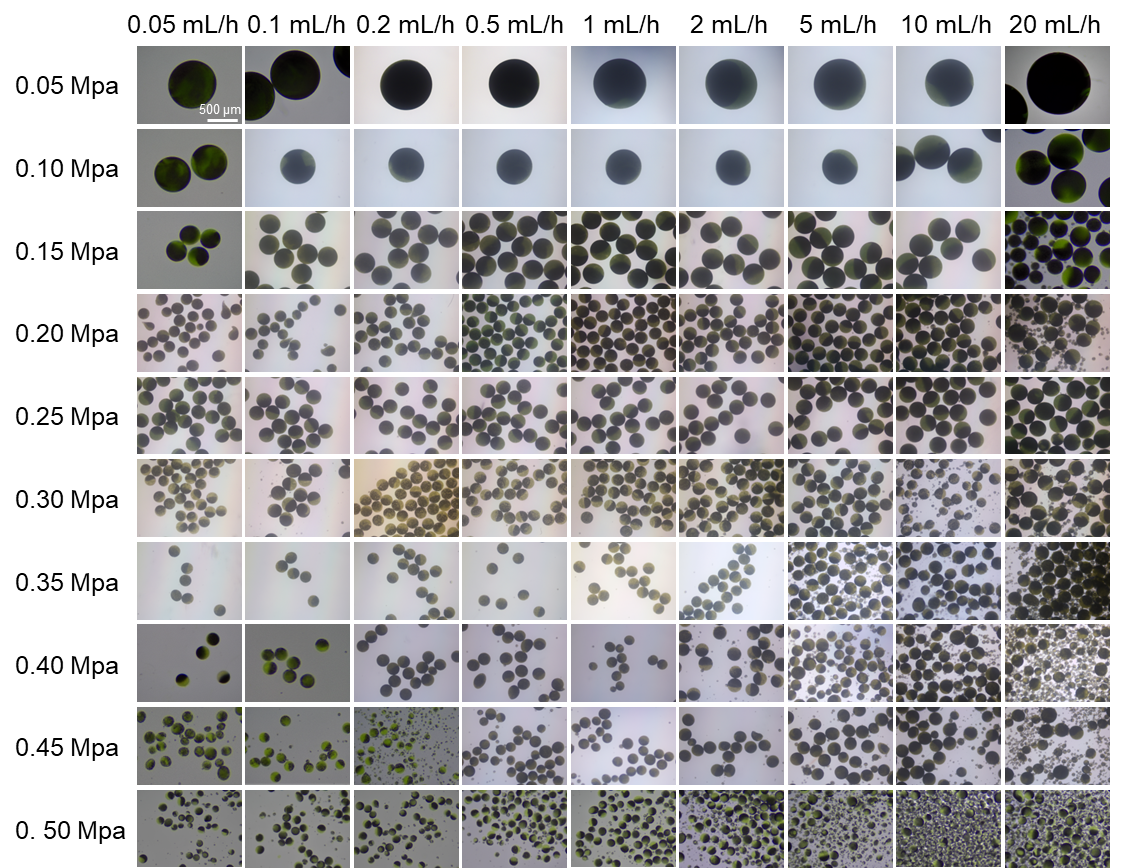


**Figure S5.** Microscope images of microgels prepared under different conditions.


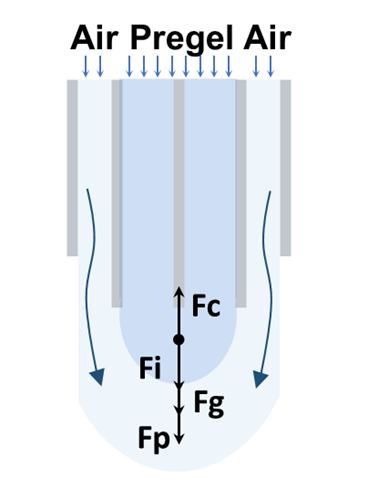


**Figure S6.** Shear fluid force analysis diagram.


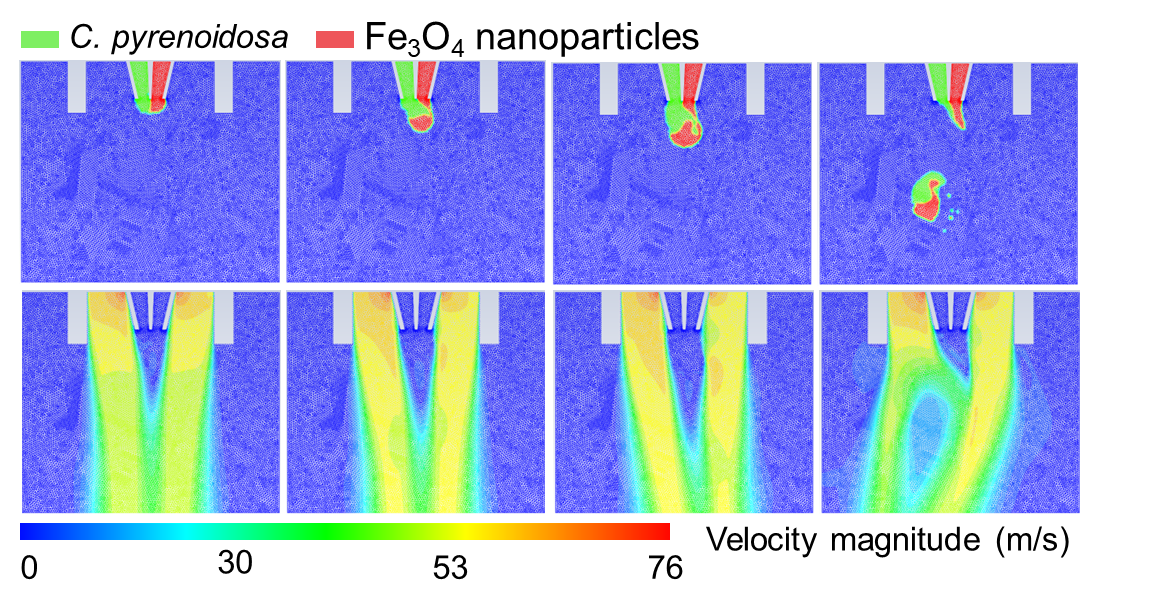


**Figure S7.** Fluid dynamic simulation of homogeneous mixtures formation.


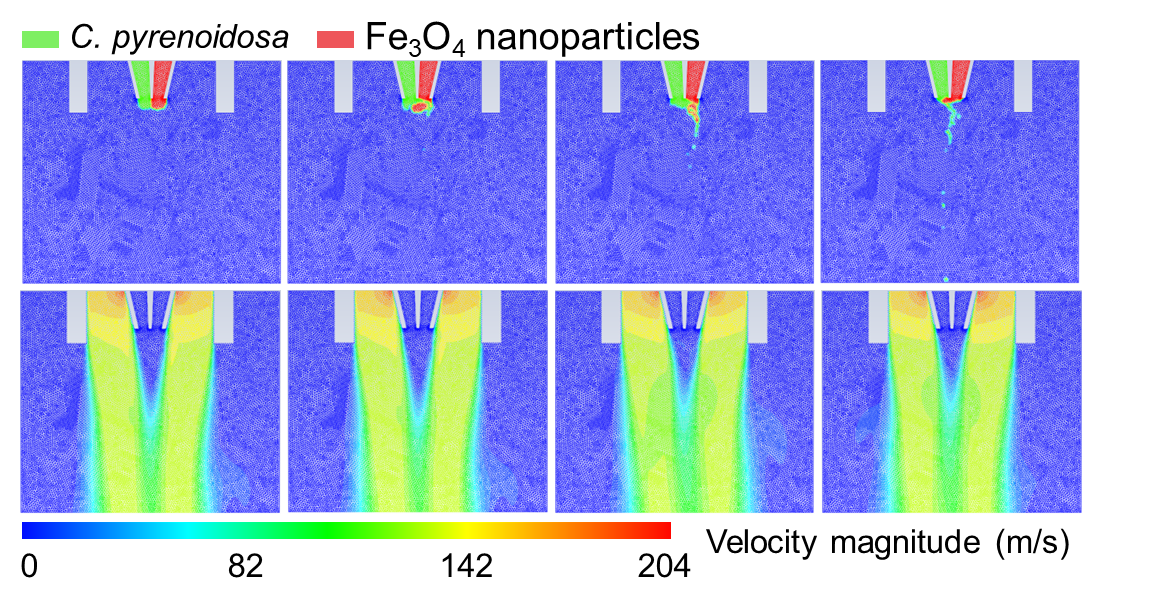


**Figure S8.** Fluid dynamic simulation of fragmented structures formation.


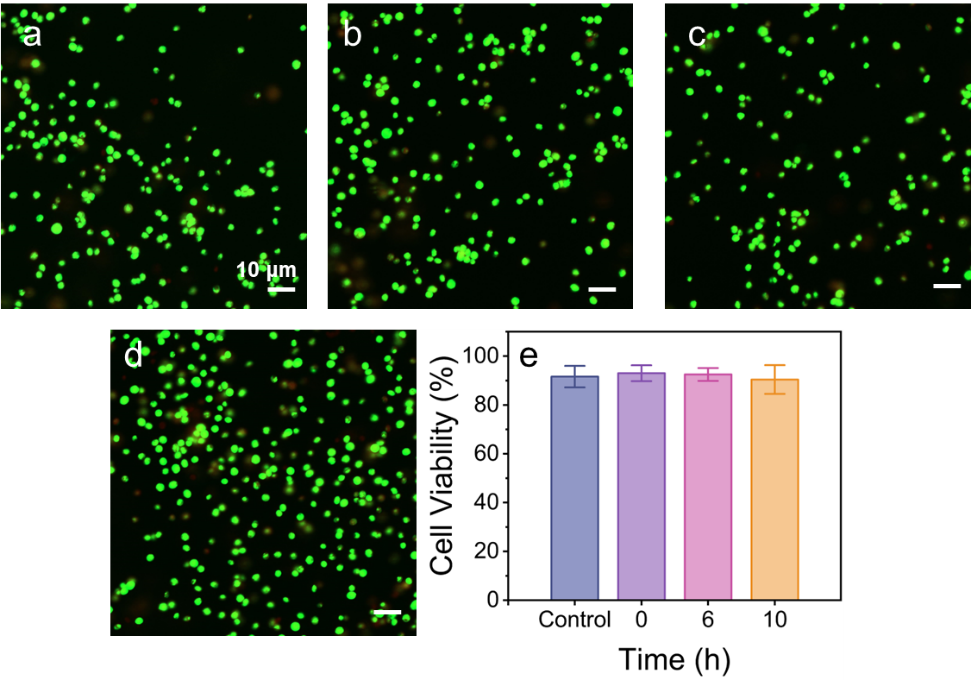


**Figure S9.** Viability of *C. pyrenoidosa* cells: (a) control, (b) pre-experiment in JMRs, (c) during operation (6 h) in JMRs, (d) post-experiment (10 h) in JMRs.


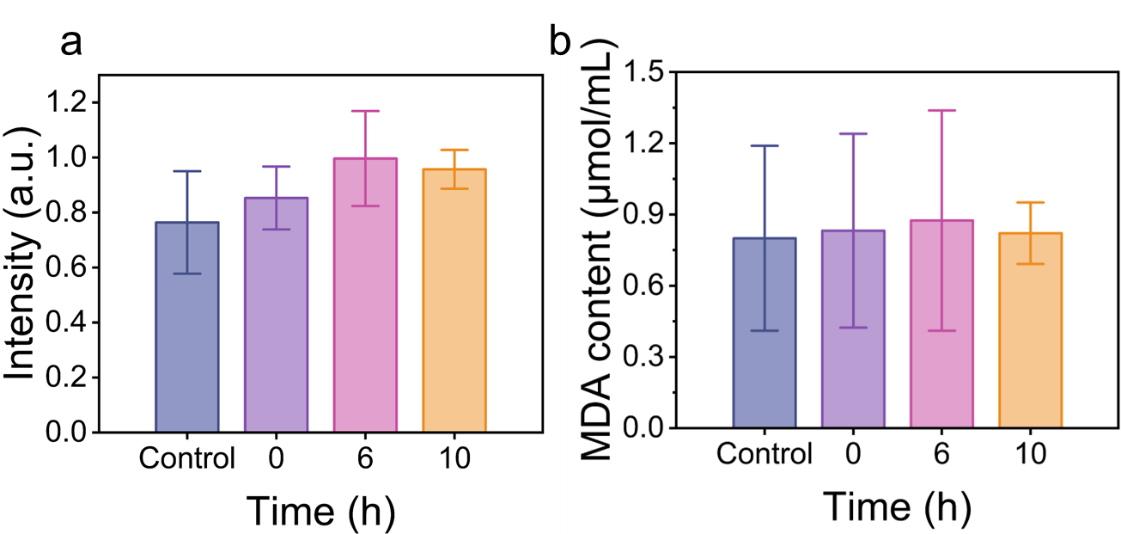


**Figure S10.** (a) The content of ROS in algal cells during the degradation process. (b) The content of MDA in algae cells during the degradation reaction process.


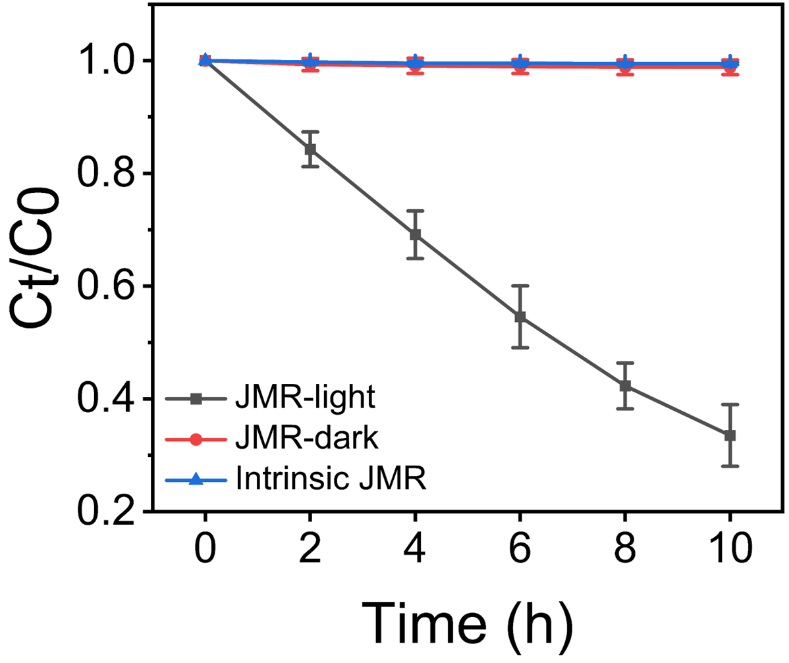


**Figure S11.** LEV removal by JMRs under light and dark conditions.

**
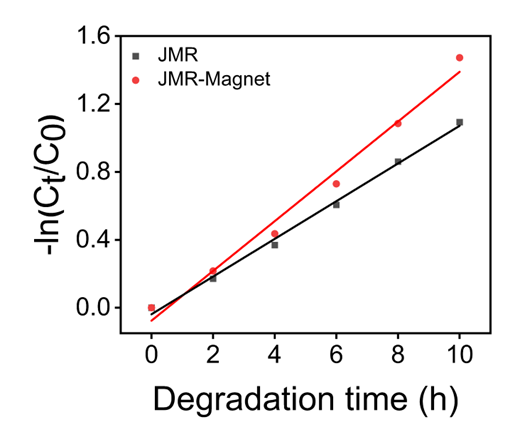
**

**Figure S12.** The degradation kinetic curve of JMRs and JMRs under rotational magnetic field.


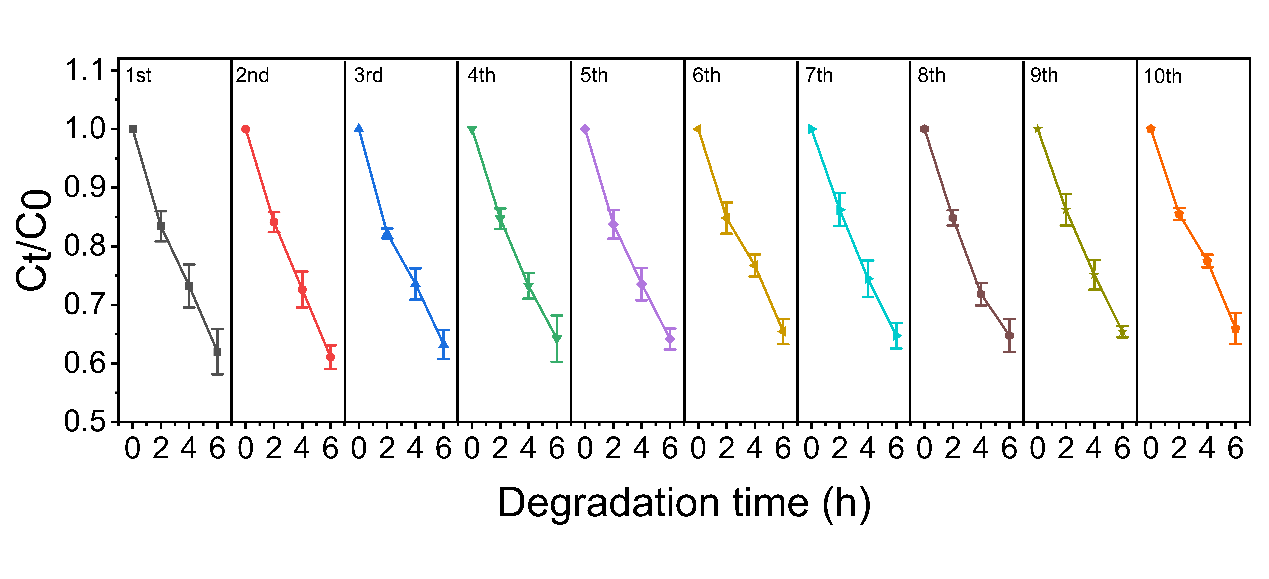


**Figure S13.** Ten consecutive LEV degradation cycles by the same batch of JMRs.


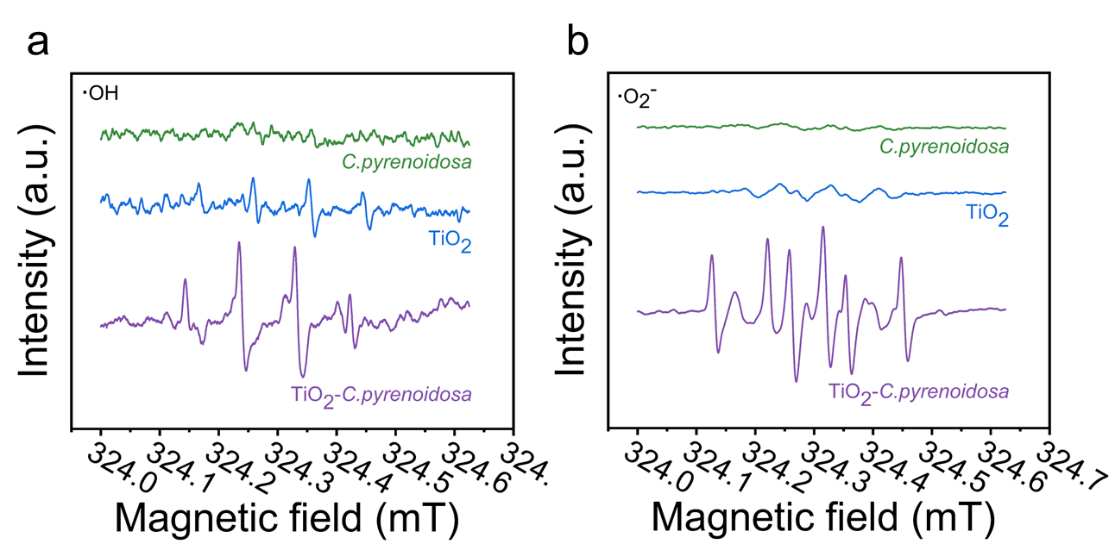


**Figure S14.** Electron spin resonance spectra of hydroxyl radicals and superoxide anions radicals in *C. pyrenoidosa*, TiO_2_, and TiO_2_-*C. pyrenoidosa* under light irradiation.

**
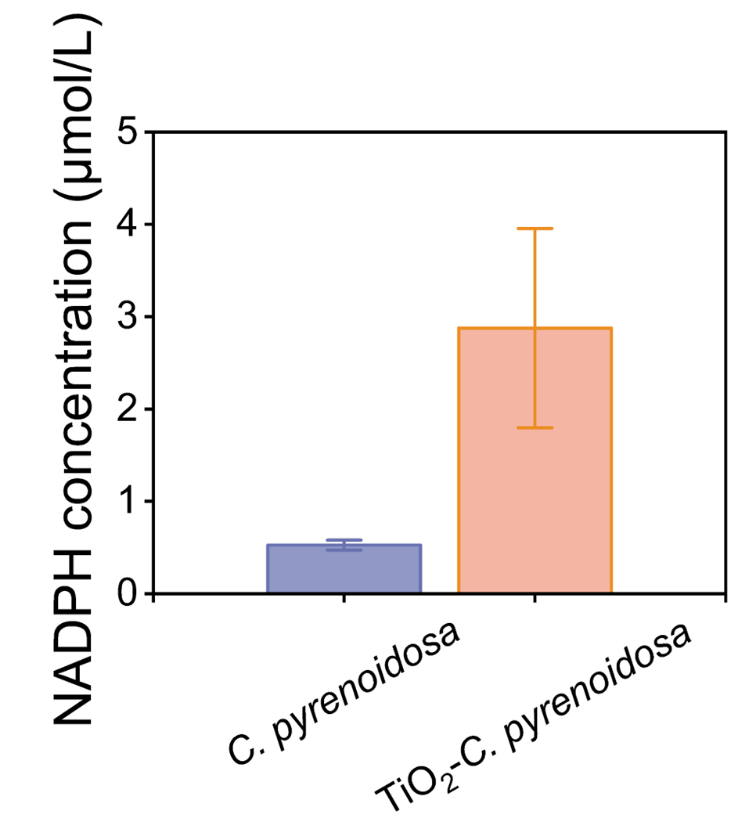
Figure S15.** Comparison of NADPH content between *C. pyrenoidosa* and TiO_2_-*C. pyrenoidosa*.


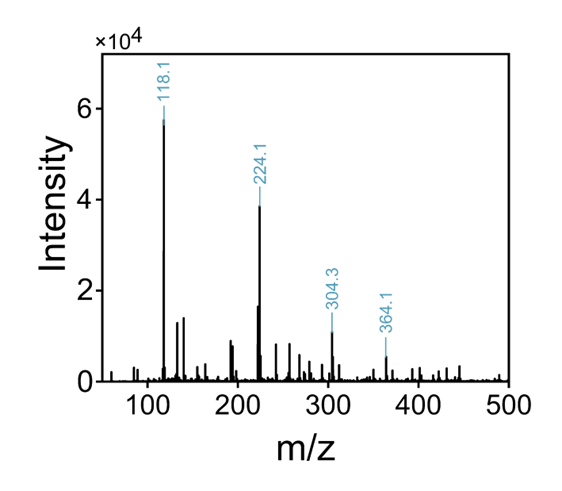


**Figure S16.** Liquid chromatography-mass spectra of LEV degradation intermediates by TiO_2_.


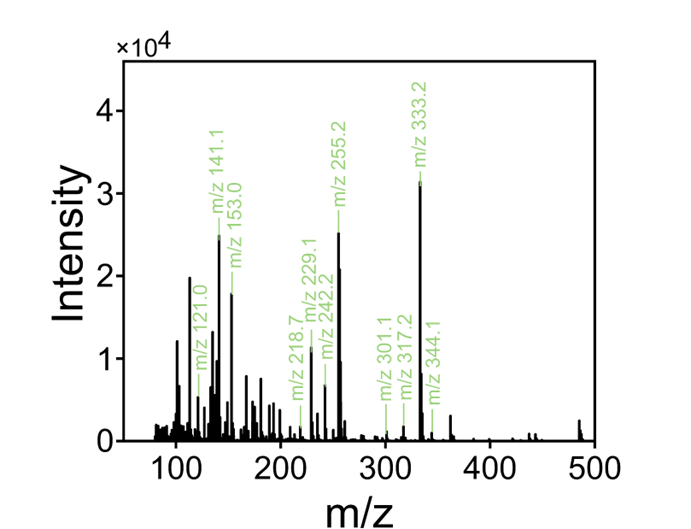


**Figure S17.** Liquid chromatography-mass spectra of LEV degradation intermediates by C.*pyrenoidosa*.

**References**

[1] Z. Zhu, T. Chen, Y. Zhu, F. Huang, K. Mu, T. Si, R. X. Xu, *Matter* **2023**, *6*, 2034-2051.

[2] Q. Hu, Y. Ren, X. Zheng, L. Hou, T. Jiang, W. Liu, Y. Tao, H. Jiang, *Microfluidics and Nanofluidics* **2019**, *23*, 13.

[3] T. Nisisako, T. Torii, T. Takahashi, Y. Takizawa, *Advanced Materials* **2006**, *18*, 1152-1156.

[4] X. Wang, Z.-H. Zhang, K.-K. Yuan, H.-Y. Xu, G.-H. He, L. Yang, J. Buhagiar, W.-D. Yang, Y. Zhang, C. S. K. Lin, H.-Y. Li, *Chemical Engineering Journal* **2023**, *465*, 142770.

[5] L. Ge, H. Deng, *Photochemical & Photobiological Sciences* **2015**, *14*, 693-699.

[6] Y. Chen, T. Xuan, M. Dong, H. Liang, G. Cai, B. Xia, J. Tang, Z. Xie, X. Zhao, *Journal of Hazardous Materials* **2025**, *500*, 140558.

[7] Z. Li, S. Li, Q. Wu, X. Gao, L. Zhu, *Journal of Hazardous Materials* **2024**, *466*, 133519.

[8] C. Kiki, A. Rashid, Y. Zhang, X. Li, T.-Y. Chen, A. B. Eloise Adéoye, P. O. Peter, Q. Sun, *Chemosphere* **2022**, *292*, 133438.

[9] G. Guo, Z. Wang, C. Lu, W. Xu, B. Lu, Y. Zhao, *Bioresource Technology* **2024**, *400*, 130668.

[10] J. Wei, Z. Wang, C. Zhao, B. Lu, H. Zhang, Y. Zhao, *Journal of Water Process Engineering* **2024**, *64*, 105683.

[11] J.-Q. Xiong, S. Ru, Q. Zhang, M. Jang, M. B. Kurade, S.-H. Kim, B.-H. Jeon, *Bioresource Technology* **2020**, *309*, 123452.
